# New findings in prognostic factor assessment for adenocarcinoma of transverse colon: a comparison study between competing-risk and COX regression analysis

**DOI:** 10.3389/fmed.2024.1301487

**Published:** 2024-01-31

**Authors:** Hongbo Su, Shuping Xie, Shanshan Wang, Liying Huang, Jun Lyu, Yunlong Pan

**Affiliations:** ^1^Department of General Surgery, The First Affiliated Hospital of Jinan University, Guangzhou, Guangdong, China; ^2^Department of Health Statistics, School of Public Health, Shanxi Medical University, Taiyuan, China; ^3^Section of Occupational Medicine, Department of Special Medicine, Shanxi Medical University, Taiyuan, Shanxi, China; ^4^Department of Clinical Research, The First Affiliated Hospital of Jinan University, Guangzhou, Guangdong, China; ^5^Guangdong Provincial Key Laboratory of Traditional Chinese Medicine Informatization, Guangzhou, Guangdong, China

**Keywords:** adenocarcinoma of transverse colon, competing-risk analysis, SEER, Cox regression, subdistribution hazard function

## Abstract

**Purpose:**

Competing-risk analysis was used to accurately assess prognostic factors for cancer-specific death in patients with adenocarcinoma of transverse colon (ATC), and the results were compared with those from a conventional Cox regression analysis.

**Materials and Methods:**

Patients diagnosed with ATC between 2000 and 2019 were selected from the Surveillance, Epidemiology, and End Results database. The crude mortality rates of patients with ATC were calculated and their differences were tested using the Gray’s test, respectively. In performing multivariate analysis, the Cox regression model and the subdistribution hazard function (SD) in competing risk analysis were utilized, respectively.

**Results:**

This study included 21,477 eligible patients. The SD model indicated that age, etc. are actual independent prognostic factors. In contrast to previous recognition, the results of the Cox regression showed false-positives for sex and Carcinoembryonic antigen, and underestimated point-estimates in the stage and American Joint Committee on Cancer stage due to competing events. A detailed comparison of treatment revealed that the larger surgical scopes were prognostic risk factors compared with the smaller scope of local tumor excision, partial colectomy, or segmental resection. Patients treated with external proton beam radiotherapy had an increased risk compared with those with no radiotherapy and internal radiotherapy.

**Conclusions:**

After comparing the results of the two methods and mitigating the significant bias introduced by Cox regression, we found independent factors that really affect the prognosis of ATC. On the other hand, in terms of ATC, a larger surgical scope and external proton beam radiotherapy may not improve the long-term survival of patients. Therefore, when faced with ATC patients, these differences should be noted and treated differently from common colorectal cancer patients. Thus, clinicians are able to give more targeted treatment plans and prognostic assessments.

## Introduction

Cancer has become the impediment to human longevity and high quality of life. According to the latest global cancer statistics from GLOBOCAN 2020, colorectal cancer ranked third in terms of new cases, with 1.93 million accounting for 10% of all new cancers, and second in terms of deaths, with 940,000 accounting for 9.4% of all deaths due to cancer in that year (1).

Transverse colon cancer has been reported to account for about 10% of colorectal cancers (2). The transverse colon is located in a special high position, in the middle and anterior part of the entire colon, between the ascending and descending colon, excluding the hepatic and splenic flexures. It has a maximum length of about 50 cm. Since the transverse colon differs from the rest of the colon in terms of embryonic development, anatomical structure, blood supply, and pathogenetic characteristics, it is necessary to clearly delineate the different segments of the colon and to provide precise and individualized treatments according to the specific characteristics of the transverse colon, which is also in line with contemporary medical concepts. However, most studies on colorectal cancer have focused on the ascending and descending colon, which have obvious differences. The transverse colon, which is the link between the two, has received little attention in research. Adenocarcinoma arises from the glandular epithelium, ducts, or secretory epithelium, and is characterized by adenoid structure formation. It is the most common clinical type of colon cancer, accounting for 90–95% of cases, and has a better prognosis than other pathological types. Research on adenocarcinoma of transverse colon (ATC) would therefore be helpful for improving the clinical outcomes.

Kaplan-Meier (KM) analyses and Cox proportional-hazards models are the classical statistical analysis methods used in investigation of the prognostic factors for colorectal cancer. However, advances in medicine and statistical methods are increasing the demand for more-accurate results. It needs to be remembered that dying from cancer is only one of the causes of death for cancer patients, since deaths due to noncancerous diseases and accidents also account for a significant proportion of the causes of death (3). It is therefore necessary to consider cancer and non-cancer factors separately when estimating patient mortality. Non-cancer deaths are often considered competing events, and their presence makes the Cox proportional-hazards model inaccurate. Therefore, when analyzing the factors affecting the prognosis of patients with ATC, using a competing-risk analysis will reduce bias and increase the accuracy of the results, thereby more accurately reflecting the true situation.

This study extracted data from the Surveillance, Epidemiology, and End Results (SEER) database on patients diagnosed with ATC (4), performed a competing-risk analysis, and compared the results with those of a Cox regression analysis. This protocol allowed for a more-accurate determination of the factors affecting the prognosis of ATC.

## Materials and methods

### Data collection and patient selection

**FIGURE 1:**
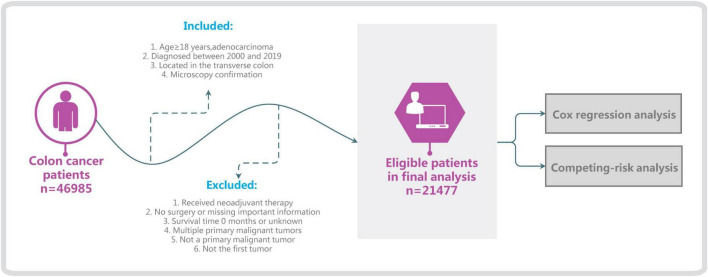
Flow chart of patient selection and study development.

Surveillance, Epidemiology, and End Results is one of the most-authoritative large oncology registry databases (5). We used SEER*Stat software (version 8.4.0) and selected the “Incidence–SEER Research Plus Data, 17 Registries, Nov 2021 Sub (2000–2019)” database, which is derived from 17 registration stations and covers 26.5% of the entire US population. Basic and medical information on patients diagnosed with ATC was extracted, specifically demographic information such as age and race, clinical information such as type of pathology and surgical modality, and survival status. The following inclusion criteria were applied: age ≥ 18 years, diagnosed between 2000 and 2019, tumor located in the transverse colon (C18.4, excluding the hepatic flexure and splenic flexure), microscopy confirmation, and adenocarcinoma (including signet-ring-cell carcinoma). The exclusion criteria were no surgery, survival time 0 months or unknown, multiple primary malignant tumors, not the first tumor, not a primary malignant tumor, or too many incomplete variables (Figure 1). Since the causes of death and survival status of patients are documented in detail in the SEER database, we classify all patients into colon cancer-specific deaths, competing events (other causes of death), and survival (6). Applying the inclusion and exclusion criteria resulted in 21,477 patients being included in this study.

### Statistical analyses

The variables in the baseline data were described using number (N) and percentage values. In the competing-risk analysis, death from ATC and death from other causes was regarded as a competitive relationship. The cumulative-risk rate was estimated in the single-factor analysis using the cumulative incidence function (CIF) described by CIF*k*(*t*) = Pr(*T* ≤ *t*, *D = k*), where function CIF*k*(*t*) represents the probability of the *k*-th event occurring before time *t* and other class events, and *D* represents the type of events that occur (7). Gray’s test was used to perform between-group comparisons (8). When no competing events exist, we used Cox regression for the multivariate analyses with the formula log[λ (*t*)] = log[λ 0 (*t*)]+ χβ ^2^, where λ 0(*t*) is the net risk and λ(*t*) is the baseline risk function; that is, the risk function when the covariate vector is 0, which can be written as λ (*t*) = λ 0(*t*)exp(χβ) (7).

When competing events are present and the deletion-independence condition is not satisfied (9), we provide the results of both the subdistribution hazard function (SD) and cause-specific (CS) hazard function belonging to competing-risk models (10). The formula used for the SD model was $\lambda_{K}^{S\,D}\,\left(t\right)={\text{lim}}_{\Delta \,t\to 0}\,\frac{P\left[t\leq T<t+\Delta t,D=k|T>t\cup \left(T<t\cap K\neq k\right)\right]}{\Delta \,t}$, where SD represents the instantaneous probability of the occurrence of the *k*-th event being observed in the individual at time *t* (11). The formula used for the CS model was $\lambda_{K}^{C\,S}\,\left(t\right)={\text{lim}}_{\Delta \,t\to 0}\,\frac{P\left(t\leq T<t+\Delta t,D=k|T\geq t\right)}{\Delta \,t}$, where CS represents the instantaneous probability of a class-*k* event being observed in the individual who did not experience any event at time *t* (12). The interpretation and usage differ between the above two models, and so the results of both models need to be provided at the same time (13). Lau et al. and Koller et al. proposed that the SD model only focus on the absolute incidence of events of interest (14, 15), and tends to be used in individual risk-prediction studies to estimate the risk and prognosis of a disease, and is suitable for establishing clinical prediction models and risk scores (4); The CS model favors etiological studies, with regression coefficients reflecting the relative effects of covariates on the increased incidence of events of interest in subjects in the event-free risk set (16). Therefore, the present study mainly adopted the conclusions from the SD model.λ_*k*_(*t*) in the formulas for the SD and CS models is the crude risk rate, which is not equal to the net risk rate when competing events are present, and so the hazards ratio (HR) value and 95% confidence interval (CI) obtained using traditional Cox analysis are biased (17). In view of this, we also compared the results of the competing-risk analysis with those of classical Cox analysis (18).

R Studio software (version 2022.02.3) was used for all statistical analyses. All statistical tests were two-sided, with a probability value of *P* < 0.05 considered to indicate statistical significance (19). The SEER database can be accessed free of charge, and this study was exempted from the need to obtain informed consent from the included patients by the institutional research committee of the First Affiliated Hospital of Jinan University.

## Results

### Clinical characteristics of patients and survival outcomes

Among the 21,477 patients with ATC who were screened, 10,867 (50.6%) died: (5,923 from ATC and 4,944 from other causes). The survival time was 64.67 ± 58.68 months.

For continuous variables, the average age of the sample was 68.08 ± 13.93 years, number of lymph nodes examined 17.78 ± 11.44, number of positive lymph nodes 1.63 ± 3.44. Table 1 lists the classification variables.

**TABLE 1:** Basic characteristics of patients in this study.

Variable	All patients (%)	Censored (%)	Concerned (%)	Competition (%)
Total number	21,477	10,610	5,923	4,944
**Race**				
White people	16,934 (78.85)	8,160 (76.91)	4,630 (78.17)	4,144 (83.82)
Black people	2,613 (12.17)	1,274 (12.01)	840 (14.18)	499 (10.09)
Other	1,930 (8.99)	1,176 (11.08)	301 (5.08)	453 (9.16)
**Sex**				
Male	10,105 (47.05)	5,126 (48.31)	2,771 (46.78)	2,208 (44.66)
Female	11,372 (52.95)	5,484 (51.69)	3,152 (53.22)	2,736 (55.34)
**Marital status**				
Married	11,316 (52.69)	6,177 (58.22)	2,945 (49.72)	2,194 (44.38)
Unmarried	10,161 (47.31)	4,433 (41.78)	2,978 (50.28)	2,750 (55.62)
**Grade**				
I	1,774 (8.26)	1,045 (9.85)	283 (4.78)	446 (9.02)
II	14,460 (67.33)	7,528 (70.95)	3,521 (59.45)	3,411 (68.99)
III	4,243 (19.76)	1,611 (15.18)	1,761 (29.73)	871 (17.62)
IV	496 (2.31)	191 (1.8)	213 (3.6)	92 (1.86)
Unknown	504 (2.35)	235 (2.21)	145 (2.45)	124 (2.51)
**Stage**				
Localized	7,801 (36.32)	4,874 (45.94)	681 (11.5)	2,246 (45.43)
Regional	10,624 (49.47)	5,253 (49.51)	2,871 (48.47)	2,500 (50.57)
Distant	3,048 (14.19)	483 (4.55)	2,370 (40.01)	195 (3.94)
Unknown	4 (0.02)	0 (0)	1 (0.02)	3 (0.06)
**AJCC**				
0	186 (0.87)	127 (1.2)	11 (0.19)	48 (0.97)
I	3,787 (17.63)	2,489 (23.46)	208 (3.51)	1,090 (22.05)
II	8,068 (37.57)	4,515 (42.55)	1,220 (20.6)	2,333 (47.19)
III	6,433 (29.95)	3,010 (28.37)	2,140 (36.13)	1,283 (25.95)
IV	2,995 (13.95)	468 (4.41)	2,343 (39.56)	184 (3.72)
Unknown	8 (0.04)	1 (0.01)	1 (0.02)	6 (0.12)
**LymphExcision**				
Yes	21,149 (98.47)	10,456 (98.55)	5,826 (98.36)	4,867 (98.44)
No	313 (1.46)	151 (1.42)	89 (1.5)	73 (1.48)
Unknown	15 (0.07)	3 (0.03)	8 (0.14)	4 (0.08)
**OthersiteSurgery**				
No	19,932 (92.81)	10,062 (94.84)	5,141 (86.8)	4,729 (95.65)
Single resection	1,151 (5.36)	400 (3.77)	612 (10.33)	139 (2.81)
Combination	81 (0.38)	27 (0.25)	43 (0.73)	11 (0.22)
Surgery, NOS	231 (1.08)	106 (1.0)	82 (1.38)	43 (0.87)
Unknown	82 (0.38)	15 (0.14)	45 (0.76)	22 (0.44)
**PrimSiteSurg**				
LPS	8,617 (40.12)	4,111 (38.75)	2,310 (39)	2,196 (44.42)
SCH	11,977 (55.77)	6,074 (57.25)	3,313 (55.93)	2,590 (52.39)
TP	504 (2.35)	287 (2.7)	142 (2.4)	75 (1.52)
Surgery, NOS	379 (1.76)	138 (1.3)	158 (2.67)	83 (1.68)
**Radiotherapy**				
No	21,300 (99.18)	10,560 (99.53)	5,814 (98.16)	4,926 (99.64)
Beam radiation	144 (0.67)	37 (0.35)	94 (1.59)	13 (0.26)
Internal radiotherapy	9 (0.04)	4 (0.04)	4 (0.07)	1 (0.02)
Radiotherapy, NOS	7 (0.03)	2 (0.02)	3 (0.05)	2 (0.04)
Unknown	17 (0.08)	7 (0.07)	8 (0.14)	2 (0.04)
**Chemotherapy**				
Yes	7,212 (33.58)	3,645 (34.35)	2,773 (46.82)	794 (16.06)
No/unknown	14,265 (66.42)	6,965 (65.65)	3,150 (53.18)	4,150 (83.94)
**MetsBone**				
Yes	35 (0.16)	3 (0.03)	31 (0.52)	1 (0.02)
No	10,992 (51.18)	7,395 (69.7)	2,217 (37.43)	1,380 (27.91)
Unknown	10,450 (48.66)	3,212 (30.27)	3,675 (62.05)	3,563 (72.07)
**MetsBrain**				
Yes	7 (0.03)	1 (0.01)	5 (0.08)	1 (0.02)
No	11,018 (51.30)	7,397 (69.72)	2,242 (37.85)	1,379 (27.89)
Unknown	10,452 (48.67)	3,212 (30.27)	3,676 (62.06)	3,564 (72.09)
**MetsLiver**				
Yes	938 (4.37)	229 (2.16)	662 (11.18)	47 (0.95)
No	10,102 (47.04)	7,168 (67.56)	1,599 (27.0)	1,335 (27.0)
Unknown	10,437 (48.60)	3,213 (30.28)	3,662 (61.83)	3,562 (72.05)
**MetsLung**				
Yes	179 (0.83)	28 (0.26)	144 (2.43)	7 (0.14)
No	10,849 (50.51)	7,370 (69.46)	2,107 (35.57)	1,372 (27.75)
Unknown	10,449 (48.65)	3,212 (30.27)	3,672 (62.0)	3,565 (72.11)
**MetsDistLN**				
Yes	55 (0.26)	25 (0.24)	29 (0.49)	1 (0.02)
No	4,421 (20.58)	3,738 (35.23)	476 (8.04)	207 (4.19)
Unknown	17,001 (79.16)	6,847 (64.53)	5,418 (91.47)	4,736 (95.79)
**MetsOther**				
Yes	4,313 (20.08)	3,680 (34.68)	429 (7.24)	204 (4.13)
No	161 (0.75)	82 (0.77)	75 (1.27)	4 (0.08)
Unknown	17,003 (79.17)	6,848 (64.54)	5,419 (91.49)	4,736 (95.79)
**TumorSize**				
≤4 cm	10,378 (48.32)	5,511 (51.94)	2,255 (38.07)	2,612 (52.83)
>4 cm	11,099 (51.68)	5,099 (48.06)	3,668 (61.93)	2,332 (47.17)
**CEA**				
Negative	7,092 (33.02)	4,130 (38.93)	1,612 (27.22)	1,350 (27.31)
Border	60 (0.28)	31 (0.29)	16 (0.27)	13 (0.26)
Positive	5,701 (26.54)	2,945 (27.76)	1,648 (27.82)	1,108 (22.41)
Unknown	8,624 (40.15)	3,504 (33.03)	2,647 (44.69)	2,473 (50.02)
**NeuroInvasion**				
Yes	1,539 (7.17)	729 (6.87)	583 (9.84)	227 (4.59)
No	12,347 (57.49)	7,411 (69.85)	2,711 (45.77)	2,225 (45.0)
Unknown	7,591 (35.34)	2,470 (23.28)	2,629 (44.39)	2,492 (50.4)

Censored: Patients who are alive; Concerned: Patients who died of ATC; Competition: Patients who died of competing events; Unmarried, including single, widowed, divorced and separated. Stage: the most basic way of categorizing how far a cancer has spread from its point of origin. AJCC: American Joint Committee on Cancer; LymphExcision: if regional lymph nodes was removed; Single resection: removal of only regional metastases, or distant lymph nodes, or distant metastases; combination: Any combination of Single resection; Surgery, NOS: Surgery was performed but method is unknown; PrimSiteSurg: Surgery of Primary Site. LPS: Local tumor excision or Partial colectomy or Segmental resection; SCH: Subtotal colectomy or hemicolectomy; TP: Total colectomy or proctocolectomy; Internal radiotherapy: Radioactive implants or Radioisotopes; Radiotherapy, NOS: Radiotherapy was performed but method is unknown; Mets: metastases; MetsBone: bone metastases; CEA: carcinoembryonic antigen.

### Univariate analyses

In the presence of competing-risk, we performed univariate analyses using the CIF and Gray’s test, with the results showing that race, marital status, grade of differentiation, summary stage, AJCC stage, surgery of primary site and other site, radiotherapy and chemotherapy status, tumor size, CEA, status of bone metastasis, brain metastasis, liver metastasis, lung metastasis, distant lymph node metastasis, other site metastasis, and neuro invasion exerted significant effects on the prognosis of ATC (*P* < 0.05). Analyzing the CIF of each variab le at 1 year, 3 years, and 5 years revealed that the CIF of almost all variables increased over time. Among the ordinal classification variables such as grade, the CIF also increased gradually with increasing degree. Detailed data are provided in Table 2. Because age at diagnosis, the number of lymph nodes examined or positive are continuous variables, they were directly included in the multivariate analyses rather than in univariate analyses.

**TABLE 2:** Univariate analysis of prognostic factors in patients with ATC.

Variable	Gray’s test	*p*-value	CIF		
			**1 year**	**3 years**	**5 years**
**Race**	**37.77**	**<0.0001**			
White people			0.107	0.218	0.267
Black people			0.102	0.249	0.321
Other			0.078	0.182	0.233
**Sex**	**0.02**	**0.8825**			
Male			0.098	0.212	0.273
Female			0.109	0.224	0.269
**Marital status**	**41.62**	**<0.0001**			
Married			0.093	0.201	0.253
Unmarried			0.117	0.238	0.290
**Grade**	**743.54**	**<0.0001**			
I			0.045	0.107	0.152
II			0.077	0.181	0.235
III			0.207	0.369	0.417
IV			0.231	0.373	0.4
Unknown			0.102	0.235	0.283
**Stage**	**7027.36**	**<0.0001**			
Localized			0.022	0.048	0.071
Regional			0.084	0.200	0.261
Distant			0.383	0.713	0.805
Unknown			0.197	0.25	0.311
**AJCC**	**7418.21**	**<0.0001**			
0			0.016	0.036	0.044
I			0.013	0.027	0.042
II			0.043	0.097	0.133
III			0.105	0.253	0.329
IV			0.385	0.718	0.811
Unknown			0.113	0.146	0.213
**LymphExcision**	**4.05**	**0.132**			
Yes			0.104	0.218	0.271
No			0.105	0.209	0.262
Unknown			0.267	0.4	0.478
**OthersiteSurgery**	**517.84**	**<0.0001**			
No			0.097	0.202	0.252
Single resection			0.197	0.454	0.537
Combination			0.198	0.451	0.534
Surgery, NOS			0.120	0.288	0.329
Unknown			0.298	0.486	0.499
**PrimSiteSurg**	**42.48**	**<0.0001**			
LPS			0.101	0.209	0.258
SCH			0.104	0.221	0.274
TP			0.107	0.235	0.302
Surgery, NOS			0.173	0.329	0.401
**Radiotherapy**	**128.16**	**<0.0001**			
No			0.103	0.216	0.268
Beam radiation			0.324	0.569	0.636
Internal radiotherapy			0.467	0.802	0.893
Radiotherapy, NOS			0.143	0.356	0.429
Unknown			0.118	0.397	0.548
**Chemotherapy**	**619.72**	**<0.0001**			
Yes			0.104	0.301	0.386
No/unknown			0.104	0.177	0.213
**MetsBone**	**290.15**	**<0.0001**			
Yes			0.771	0.9	0.95
No			0.082	0.185	0.237
Unknown			0.124	0.246	0.298
**MetsBrain**	**133.84**	**<0.0001**			
Yes			0.857	0.862	0.875
No			0.084	0.187	0.23866
Unknown			0.124	0.247	0.29829
**MetsLiver**	**1,829**	**<0.0001**			
Yes			0.343	0.707	0.8069
No			0.060	0.139	0.18581
Unknown			0.123	0.246	0.29749
**MetsLung**	**654.06**	**<0.0001**			
Yes			0.473	0.830	0.90761
No			0.078	0.177	0.22818
Unknown			0.124	0.246	0.29798
**MetsDistLN**	**142.06**	**<0.0001**			
Yes			0.296	0.818	0.874
No			0.069	0.158	0.235
Unknown			0.112	0.228	0.279
**MetsOther**	**245.65**	**<0.0001**			
Yes			0.283	0.734	0.875
No			0.064	0.145	0.237
Unknown			0.112	0.228	0.279
**TumorSize**	**402.69**	**<0.0001**			
≤4 cm			0.067	0.158	0.209
>4 cm			0.139	0.275	0.329
**CEA**	**71.57**	**<0.0001**			
Negative			0.085	0.184	0.235
Border			0.124	0.230	0.276
Positive			0.114	0.246	0.301
Unknown			0.113	0.228	0.279
**NeuroInvasion**	**252.55**	**<0.0001**			
Yes			0.149	0.342	0.425
No			0.085	0.186	0.234
Unknown			0.126	0.245	0.296

CIF, cumulative incidence function.

### Multivariate analyses

All variables that were statistically significant in the univariate analyses (*P* < 0.05) were entered into a Cox regression analysis and a competing-risk analysis for the multivariate analyses (20). Since sex and regional lymphadenectomy were important demographic and surgical information, respectively, they also needed to be added to the multivariate analyses even though they were not statistically significant in univariate analyses (21).

Both the Cox regression analysis and the SD model indicated that age, race, grade, stage, AJCC, lymph node excision status, surgery of primary site, radiotherapy and chemotherapy status, marital status, tumor size, the number of lymph nodes examined or positive, status of bone metastasis, lung metastasis, liver metastasis, and neuro invasion were independent factors affecting the prognosis of ATC, and the results for each subgroup were basically consistent in different models. When analyzing the surgery of primary site, the HR values of subtotal colectomy or hemicolectomy, and total colectomy or proctocolectomy gradually increased with the expansion of surgical scope. The same phenomenon was observed in patients who received external proton-beam radiation. Beam radiation became a risk factor, but there was no statistically significant difference between those who did and did not receive internal radiotherapy (22). It was particularly interesting that being female and CEA-positive were risk factors in the Cox regression analysis. But in the SD model, there was no statistical difference between male and female and CEA-positive was also not a risk factor. There was no significant difference between patients with CEA-borderline and those who were CEA-negative. Most of the results from the CS model were consistent with those from the SD model. Since the CS model is often used to explore etiological issues, it only played an auxiliary role in the competing-risk analysis of this study, and so is not considered in detail (see Table 3 for more details).

**TABLE 3:** Multivariate analysis of 3 models of prognostic factors in patients with ATC.

	COX model				SD model				CS model			
**Prognostic factors**	***p*-value**	**HR**	**95% CI**		***p*-value**	**HR**	**95% CI**		***p*-value**	**HR**	**95% CI**	
**Age**	<0.0001	1.04	1.038	1.042	<0.0001	1.012	1.01	1.015	<0.0001	1.018	1.016	1.021
**Sex**												
Male	Reference				Reference				Reference			
Female	<0.0001	0.828	0.796	0.862	0.0897	0.954	0.903	1.007	0.0112	0.933	0.884	0.984
**Race**												
White people	Reference				Reference				Reference			
Black people	0.0009	1.105	1.042	1.172	0.0157	1.099	1.018	1.187	0.0108	1.103	1.023	1.19
Other	<0.0001	0.797	0.74	0.859	0.0255	0.893	0.808	0.986	0.0038	0.866	0.786	0.955
**Marital status**												
Married	Reference				Reference				Reference			
Unmarried	<0.0001	1.266	1.216	1.318	0.0032	1.088	1.029	1.15	<0.0001	1.143	1.083	1.207
**Grade**												
I	Reference				Reference				Reference			
II	0.15	1.058	0.98	1.144	0.1467	1.094	0.969	1.235	0.1698	1.089	0.964	1.231
III	<0.0001	1.29	1.185	1.404	<0.0001	1.487	1.307	1.693	<0.0001	1.508	1.326	1.714
IV	<0.0001	1.518	1.324	1.739	<0.0001	1.652	1.368	1.996	<0.0001	1.735	1.448	2.079
Unknown	0.0074	1.212	1.053	1.395	0.0002	1.483	1.203	1.828	<0.0001	1.513	1.238	1.851
**Stage**												
Localized	Reference				Reference				Reference			
Regional	<0.0001	1.186	1.109	1.267	<0.0001	1.489	1.326	1.672	<0.0001	1.525	1.358	1.712
Distant	<0.0001	1.848	1.377	2.481	<0.0001	3.149	2.104	4.714	<0.0001	3.25	2.279	4.634
Unknown	0.8369	0.854	0.191	3.828	<0.0001	3800.637	442.08	32674.75	0.9062	4105.527	0	5.01E+63
**AJCC**												
0	Reference				Reference				Reference			
I	0.1761	1.199	0.922	1.559	0.9826	1.007	0.555	1.826	0.9167	1.033	0.562	1.898
II	0.0065	1.441	1.107	1.875	0.0105	2.157	1.197	3.887	0.0077	2.263	1.241	4.129
III	<0.0001	2.054	1.568	2.691	<0.0001	4.297	2.368	7.796	<0.0001	4.77	2.598	8.756
IV	<0.0001	4.435	2.989	6.58	<0.0001	7.54	3.705	15.347	<0.0001	9.116	4.555	18.246
Unknown	0.0652	2.983	0.934	9.529	<0.0001	0.001	0	0.003	0.9199	0.001	0	9.97E+56
**LymphExcision**												
Yes	Reference				Reference				Reference			
No	0.0276	1.191	1.019	1.392	0.0255	1.275	1.03	1.579	0.0067	1.337	1.084	1.65
Unknown	0.0024	2.462	1.375	4.408	<0.0001	4.157	2.427	7.121	<0.0001	4.51	2.231	9.117
**PrimSiteSurg**												
LPS	Reference				Reference				Reference			
SCH	0.017	1.05	1.009	1.093	0.0004	1.108	1.047	1.172	<0.0001	1.126	1.065	1.189
TP	<0.0001	1.358	1.183	1.559	0.0001	1.444	1.198	1.741	<0.0001	1.496	1.26	1.776
Surgery, NOS	0.0417	1.146	1.005	1.305	0.0989	1.161	0.972	1.386	0.0129	1.229	1.045	1.447
**Radiotherapy**												
no	Reference				Reference				Reference			
Beam radiation	0.0019	1.367	1.122	1.667	<0.0001	1.515	1.239	1.854	0.0002	1.489	1.206	1.839
Internal radiotherapy	0.8319	1.1	0.455	2.658	0.6201	0.834	0.408	1.707	0.8044	0.883	0.329	2.369
Radiotherapy, NOS	0.388	0.679	0.282	1.636	0.8544	0.868	0.191	3.947	0.6929	0.796	0.256	2.475
Unknown	0.9054	0.963	0.517	1.793	0.5205	1.315	0.57	3.033	0.5833	1.215	0.606	2.436
**Chemotherapy**												
Yes	Reference				Reference				Reference			
No/unknown	<0.0001	1.471	1.4	1.545	<0.0001	1.244	1.166	1.327	<0.0001	1.461	1.375	1.552
**LymphExamed**	<0.0001	0.983	0.981	0.985	<0.0001	0.983	0.98	0.986	<0.0001	0.979	0.976	0.982
**LymphPositive**	<0.0001	1.066	1.061	1.072	<0.0001	1.059	1.052	1.066	<0.0001	1.069	1.063	1.075
**MetsBone**												
Yes	Reference				Reference				Reference			
No	0.0471	0.679	0.463	0.995	0.034	0.668	0.46	0.97	0.0468	0.673	0.455	0.994
Unknown	0.1702	0.598	0.287	1.247	0.8796	0.942	0.432	2.054	0.3958	0.724	0.344	1.525
**MetsLiver**												
Yes	Reference				Reference				Reference			
No	<0.0001	0.793	0.72	0.873	0.0004	0.82	0.735	0.915	<0.0001	0.813	0.732	0.903
Unknown	0.4724	0.844	0.531	1.34	0.8765	0.956	0.539	1.695	0.4963	0.846	0.522	1.371
**MetsLung**												
Yes	Reference				Reference				Reference			
No	<0.0001	0.703	0.591	0.837	0.0091	0.751	0.606	0.931	<0.0001	0.694	0.58	0.831
Unknown	0.1152	0.666	0.402	1.104	0.0874	0.564	0.292	1.088	0.0265	0.553	0.327	0.933
**TumorSize**												
≤4 cm	Reference				Reference				Reference			
>4 cm	<0.0001	1.138	1.09	1.187	<0.0001	1.154	1.088	1.223	<0.0001	1.194	1.126	1.265
**CEA**												
Negative	Reference				Reference				Reference			
Border	0.3052	1.211	0.84	1.748	0.35	1.259	0.777	2.039	0.4315	1.219	0.744	1.999
Positive	0.0002	1.104	1.048	1.163	0.094	1.063	0.99	1.143	0.0171	1.088	1.015	1.166
Unknown	0.0024	1.08	1.028	1.134	0.3634	1.033	0.964	1.106	0.0836	1.062	0.992	1.136
**NeuroInvasion**												
Yes	Reference				Reference				Reference			
No	0.0002	0.863	0.799	0.931	0.0314	0.896	0.81	0.99	0.0038	0.872	0.795	0.957
Unknown	0.0006	0.863	0.794	0.939	0.1045	0.91	0.811	1.02	0.0307	0.889	0.799	0.989

HR, hazard radio; CI, confidence interval; CS, cause-specific hazard; SD, subdistribution hazard function.

## Discussion

Colorectal cancer is becoming more and more common. In terms of anatomical sites, there have been many studies about the ascending colon, descending colon, and sigmoid colon, while research into the transverse colon is rare, and so many aspects of this part are still uncertain. Adenocarcinoma is one of the most common types. Therefore, it is necessary to perform further related investigations.

The findings of the present study were consistent with previous studies finding that age, etc., are independent factors influencing the prognosis (23). The HR values and *p*-values for tumor size were consistent in the three models, which also confirms the results of previous studies, indicating that patients with tumors larger than 4 cm have a relatively poorer prognosis (24). There is general agreement that racial disparities in health insurance and medical care result in black people having a higher risk. The conclusion that other races have a better prognosis than white people in this study may differ from the results of some other studies, which may be caused by genetic differences under similar medical conditions. The grade of differentiation also significantly affected the prognosis. In addition to moderate differentiation (grade II), grades III and IV had significant effects on the survival rate compared with well differentiation (grade I). Some previous studies on a similar topic have applied competing-risk analyses, and found a significant difference between grades I and II in SD and CS models, but not in the Cox model, which can be interpreted as a false-negative result (7). Whereas, the results obtained in the present study for the three models indicated no significant difference between grades II and I. We believe that this discrepancy is attributable to differences in the characteristics or sizes of the selected samples, and it could also be due to inherent characteristics of ATC itself.

The statistical results of above variables are consistent in the range of *P*-values in the three models of this study. The correlation direction of risk factors and results was consistent. HR and 95% CI in Cox regression were slightly lower but still basically similar. It is worth noting that the point estimation and interval estimation of some factors differed markedly among the three models. For example, Cox regression analysis underestimated the risk of each level of AJCC by almost half, compared with the HR values for the SD and CS models, and the 95% CI were also correspondingly lower. This echoes what other researchers have found (25). Compared with the results of Cox regression and competing-risk analysis in the “stage-distant” group, the degree of risk underestimation was surprisingly similar. Obviously, due to the existence of competing events (26), Cox regression deviates markedly in both point estimation and interval estimation, whereas the results of the competing-risk analysis are more accurate (27).

All of the independent related factors mentioned above have been widely recognized in previous studies. Our study not only further supports these conclusions, but also has produced some new findings by using competing-risk analysis. Many previous studies of colorectal cancer considered being female as a protective factor, and the present Cox regression analysis produced the same result. Based on the large population, this was not the case for the SD model, which indicated no difference in prognosis between males and females with ATC. Our analysis suggests that the incidence is slightly lower in females than males, but no difference was found in prognosis. This is consistent with the findings of Cheung et al. (28). We therefore consider this to be a false-positive result caused by competing events.

In terms of treatment, surgery has previously been considered a protective factor, but differences among specific surgical methods have not been discussed. Based on the records in the SEER database, we divided the surgical procedures into three categories. For all three models, SCH and TP with a larger surgical scope did not appear to benefit patients with ATC, but the risk increased as the surgical scope expanded relative to LPS covering a smaller scope. A larger surgical scope may tend to be applied more when the disease advanced, and is also associated with a greater tissue damage, resulting in a poor prognosis (29). Compared with the ascending colon and descending colon, the operation method of transverse colon is not so fixed, since a small deviation of the scope of hemicolectomy forward or backward can involve the ascending or descending colon (30). Sometimes the prognosis varies markedly depending on whether or not the middle colon artery is preserved (31). The results of the present competing-risk analysis further confirmed the results of the Cox regression analysis, hence making the results more robust and reliable.

The same was true for the variable of radiotherapy. Previous studies have only cared about whether patients received radiotherapy or not, and their conclusions are controversial. Some researchers have suggested that radiotherapy improves survival (32). However, studies that found radiotherapy to not be beneficial for long-term survival did not explore the underlying reasons. In contrast, the present study analyzed detailed information on radiotherapy, and found that the increased risk is mainly due to the use of external proton beam, which is the most common type of external radiotherapy, while internal radiotherapy such as I^125^ did not affect the prognosis. This may be due to external radiotherapy killing some tumor cells, reducing the tumor load, relieving symptoms, and improving short-term survival. However, due to the side effects of external radiotherapy, such as perforation, bleeding, and pancytopenia, and the generally late stage of patients who need radiotherapy, the long-term prognosis might not be improved (33). The present competing-risk analysis confirmed the results of the Cox regression analysis again.

Carcinoembryonic antigen is considered to be an important tumor marker for the diagnosis and monitoring of recurrence and metastasis, which has become a broad clinical consensus, but it often presents false-positive or false-negative results. Regarding the effect of CEA on prognosis, there is still a lot of controversy. The Cox regression analysis and the competing-risk analysis performed in this study produced different results. Elevated CEA was considered a risk factor for a poor prognosis in Cox regression, but no such difference was observed in the SD model. CEA assessment plays an important role in postoperative follow-up, in terms of detecting recurrence and metastasis early, thus improving the excision rate. However, this does not mean a higher or lower survival rate. It often needs to be combined with other indicators to evaluate the prognosis. Relevant studies by Ohlsson et al. (34) in Sweden and Kjeldsen et al. (35) in Denmark have confirmed this conclusion. Based on our results, particularly the findings from the SD model, it is evident that CEA cannot be used for prognostic prediction. This aligns with the prevailing views among most clinical experts and scholars. The clinical utility of CEA is therefore limited to the two points mentioned above. Our results once again validate the conclusions drawn by the majority of scholars. Relying solely on the results of Cox regression, however, can lead to significant errors. The Cox regression incorrectly estimates *P-*values due to the existence of competing events, and the results of competing-risk analysis will be more consistent with reality. It is well known that the location of colorectal cancer is closely related to the biological characteristics, genetic and epigenetic characteristics, pathological characteristics, and the prognosis of tumor cells (36). Tumors vary by site over time, and various features of tumors will also change gradually along the colon segment.

The transverse colon is quite special because it is located between the ascending colon and descending colons, traverses the upper abdomen, and entangled by the transverse mesocolon, which is an internal peritoneal organ. It abuts many important organs which differ from that of the ascending colon and descending colon. In addition, it is mainly supplied by the middle colon artery, and the type of surgical method is directly related to whether the artery is preserved or not. Regional lymph nodes are mainly distributed along the middle colonic artery (37), which makes lymph node dissection in this region a considerable surgical challenge. Therefore, the above-mentioned new findings in this study cannot only be attributed to the advantages of competing-risk analysis, but also reflect potential differences between ATC and cancers in other parts of the colon.

The classic KM and Cox methods rely on the assumption that censoring time and failure time are independent (38); that is, there is a single endpoint without competing events. However, in reality, clinical research data often contains a substantial amount of right-censored data due to loss of follow-up and other reasons, leading to multiple outcomes with competitive relationships. Using classical KM for univariate analyses without considering these biases will overestimate cumulative mortality, while using Cox regression for multivariate analyses can lead to further biases. (39). Our study has confirmed this theory. Some Cox regression results may have false-positives and biased effect estimates due to serious bias at a proportion of competing events exceeding 10%, with lower proportions also potentially resulting in false-positive or false-negative results (7). Competing-risk analysis can overcome these shortcomings by establishing the dependence between correlation degree and covariates, which can better and more accurately explain the effect of covariates and standardize the distribution function for different types of competing risk (7). It is noteworthy that the HR and 95% CI values obtained from the SD model were similar to those derived from the CS model, with consistent direction of associations and effect sizes that are basically consistent with the theory of SD ≤ CS; however, some results were inconsistent, which is also consistent with some studies. For instance, *P*-values for some factors in the SD and CS models were consistent, while others were not. This highlights the importance of obtaining results using both models in a competing-risk analysis, which helps to further differentiate the role of risk factors (39). Broadly speaking, CS addresses upstream epidemiological questions related to disease etiology, while SD focuses on downstream clinical event rates. The SD model is primarily used for prognostic analysis, risk scoring, and clinical prediction modeling. Therefore, it is crucial to use competing-risk analysis when analyzing the prognostic risk factors of patients where competing events are present (40).

As far as we know, this study is the first to report on the competing-risk analysis of prognostic factors for ATC, specifically using the largest number of samples and variables. It is based on the high-quality and large SEER database (41), allowing for the identification of accurate prognostic factors for specific diseases like ATC. Moreover, it provides valuable insights for clinicians in assessing prognosis and avoiding harmful treatment strategies. Additionally, it serves as a reminder to researchers about the significant inaccuracies associated with the use of Cox regression. The study also has some limitations. First, we only selected patients from 2000 to 2019, which may have introduced bias due to the short time span. Second, although the SEER database contains a significant amount of variable information, it does not cover all information that may affect patient survival, such as gene expression (42). Thus, further research is still needed to address these limitations.

## Conclusion

Upon comparative analysis of the two methodologies, it provided conclusive evidence that age, etc. are the actual prognostic factors for ATC. However, sex and CEA do not qualify as independent prognostic factors. When analyzing prognostic factors with multiple endpoints, competing-risk analysis is more accurate and reliable than COX regression, which is prone to significant bias in the presence of competing events. Additionally, larger surgical scopes and external proton-beam radiotherapy may not improve long-term survival outcomes for patients with ATC. Therefore, clinicians should take note of these differences when treating ATC patients and may need to approach them differently from common cases of colorectal cancer. This study specifically examined ATC patients in detail, in contrast to previous crude analyses of prognostic factors for colorectal cancer. These results will contribute to a deeper understanding of ATC, as well as to diagnostic, therapeutic, and clinical decision-making processes.

## Data availability statement

Publicly available datasets were analyzed in this study. This data can be found here: seer.cancer.gov/.

## Ethics statement

Ethical approval was not required for the study involving humans in accordance with the local legislation and institutional requirements. Written informed consent to participate in this study was not required from the participants or the participants’ legal guardians/next of kin in accordance with the national legislation and the institutional requirements.

## Author contributions

HS: Data curation, Formal analysis, Software, Visualization, Writing—original draft, Writing—review and editing. SX: Software, Writing—review and editing. SW: Writing—review and editing. LH: Investigation, Visualization, Writing—review and editing. JL: Conceptualization, Writing—review and editing. YP: Conceptualization, Writing—review and editing.
